# 4-Bromo-*N*-(di-*n*-propyl­carbamothioyl)­benzamide

**DOI:** 10.1107/S1600536809003511

**Published:** 2009-02-04

**Authors:** Gün Binzet, Ulrich Flörke, Nevzat Külcü, Hakan Arslan

**Affiliations:** a Department of Chemistry, Faculty of Arts and Science, Mersin University, Mersin, TR 33343, Turkey; b Department of Chemistry, University of Paderborn, Paderborn D-33098, Germany; c Department of Natural Sciences, Fayetteville State University, Fayetteville, NC 28301, USA; d Department of Chemistry, Faculty of Pharmacy, Mersin University, Mersin, TR 33169, Turkey

## Abstract

The synthesis of the title compound, C_14_H_19_BrN_2_OS, involves the reaction of 4-bromo­benzoyl chloride with potassium thio­cyanate in acetone followed by condensation of the resulting 4-bromo­benzoyl isothio­cyanate with di-*n*-propyl­amine. Typical thio­urea carbonyl and thio­carbonyl double bonds, as well as shortened C—N bonds, are observed in the title compound. The short C—N bond lengths in the centre of the mol­ecule reveal the effects of resonance in this part of the mol­ecule. The asymmetric unit of the title compound contains two crystallographically independent mol­ecules, *A* and *B*. There is very little difference between the bond lengths and angles of these mol­ecules. In mol­ecule *B*, one di-*n*-propyl group is twisted in a −anti­periplanar conformation with C—C—C—H = −179.1 (3)° and the other adopts a −synclinal conformation with C—C—C—H = −56.7 (4)°; in mol­ecule *A* the two di-*n*-propyl groups are twisted in + and −anti­periplanar conformations, with C—C—C—H = −179.9 (3) and 178.2 (3)°, respectively. In the crystal, the mol­ecules are linked into dimeric pairs *via* pairs of N—H⋯S hydrogen bonds.

## Related literature

For synthesis, see: Özer *et al.* (2009 ▶); Mansuroğlu *et al.* (2008 ▶); Uğur *et al.* (2006 ▶); Arslan *et al.* (2003*b* ▶, 2006 ▶), and references therein. For general background, see: Koch (2001 ▶); El Aamrani *et al.* (1998 ▶, 1999 ▶); Arslan *et al.* (2006 ▶, 2007*a* ▶,*b* ▶). For related compounds, see: Khawar Rauf *et al.* (2009*a* ▶,*b* ▶,*c* ▶,*d* ▶); Arslan *et al.* (2003*a* ▶, 2004 ▶). For bond-length data, see: Allen *et al.* (1987 ▶).
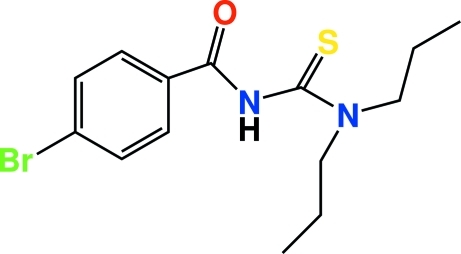


## Experimental

### 

#### Crystal data

C_14_H_19_BrN_2_OS
*M* *_r_* = 343.28
Monoclinic, 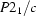

*a* = 21.104 (3) Å
*b* = 9.6940 (12) Å
*c* = 16.208 (2) Å
β = 108.956 (3)°
*V* = 3135.9 (7) Å^3^
*Z* = 8
Mo *K*α radiation
μ = 2.75 mm^−1^
*T* = 120 (2) K
0.48 × 0.18 × 0.17 mm

#### Data collection

Bruker SMART APEX diffractometer
Absorption correction: multi-scan (*SADABS*; Sheldrick, 2004 ▶) *T*_min_ = 0.352, *T*_max_ = 0.652
27091 measured reflections
7470 independent reflections
4686 reflections with *I* > 2σ(*I*)
*R*_int_ = 0.074

#### Refinement

*R*[*F* ^2^ > 2σ(*F* ^2^)] = 0.043
*wR*(*F* ^2^) = 0.094
*S* = 0.97
7470 reflections
340 parameters
2 restraints
H atoms treated by a mixture of independent and constrained refinement
Δρ_max_ = 1.70 e Å^−3^
Δρ_min_ = −0.71 e Å^−3^

### 

Data collection: *SMART* (Bruker, 2002 ▶); cell refinement: *SAINT* (Bruker, 2002 ▶); data reduction: *SAINT*; program(s) used to solve structure: *SHELXS97* (Sheldrick, 2008 ▶); program(s) used to refine structure: *SHELXL97* (Sheldrick, 2008 ▶); molecular graphics: *SHELXTL* (Sheldrick, 2008 ▶); software used to prepare material for publication: *SHELXTL*.

## Supplementary Material

Crystal structure: contains datablocks I, global. DOI: 10.1107/S1600536809003511/at2717sup1.cif

Structure factors: contains datablocks I. DOI: 10.1107/S1600536809003511/at2717Isup2.hkl

Additional supplementary materials:  crystallographic information; 3D view; checkCIF report

## Figures and Tables

**Table 1:** Hydrogen-bond geometry (Å, °)

*D*—H⋯*A*	*D*—H	H⋯*A*	*D*⋯*A*	*D*—H⋯*A*
N11—H1⋯S2	0.896 (15)	2.600 (19)	3.460 (3)	161 (3)
N21—H2⋯S1	0.899 (14)	2.566 (17)	3.452 (3)	169 (2)

**Fig. 1.**
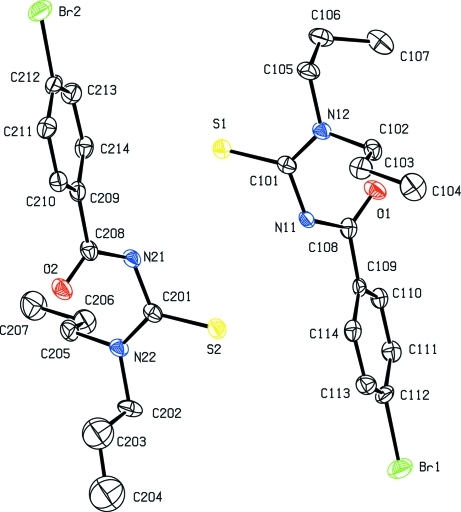
The molecular structure of (I). Displacement ellipsoids are drawn at the 50% probability level.

**Fig. 2.**
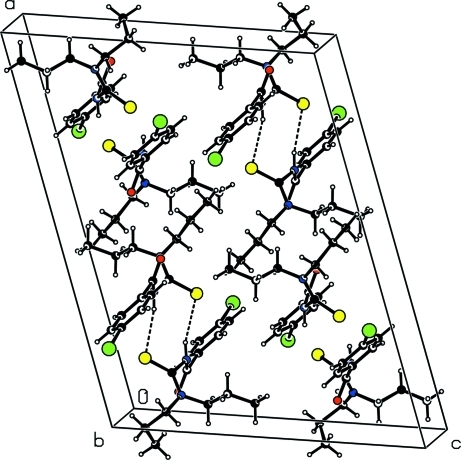
A packing diagram for (I). Hydrogen bonds are shown as dashed lines.

**Fig. 3.**
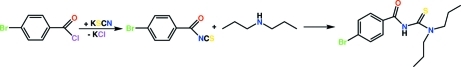
The formation of the title compound.

## supplementary crystallographic information

### Comment

Thiourea derivative ligands and their metal complexes have been one of the highlights in coordination chemistry. The thiourea ligands which contain carbonyl and thiocarbonyl groups are used as reactant for extraction of some transition metal ions (Koch, 2001; El Aamrani *et al.*, 1998, 1999). The structures of thiourea derivatives and its metal complexes have been determined during the last years. The title compound derivative acts as a bidentate ligand coordinating through the S atom and the O atom.

The similar structures of these derivatives palladium, nickel, cobalt, and copper complexes and ligands have been determined in previous studies (Özer *et al.*, 2009; Arslan *et al.*, 2003*b*, 2006; Mansuroğlu *et al.*, 2008; Uğur *et al.*, 2006). The title compound, 4-bromo-*N*-(di-*n*-propylcarbamothioyl)benzamide, (I), is another example of our newly synthesized thiourea derivatives that contains both aryl and alkyl groups.

The molecular structure of the title compound is depicted in Fig. 1. The asymmetric unit of the title compound contains two crystallographically independent molecules A (atom numbering 1xx) and B (2xx). There is very little difference between the bond lengths and angles of these molecules.

The typical thiourea carbonyl and thiocarbonyl double bonds as well as shortened C—N bond lengths are observed in the title compound. These bond lengths in the title compound are comparable to those of related structures; 1-(4-chlorobenzoyl)-3-(2,4,6-trichlorophenyl)thiourea (Khawar Rauf *et al.*, 2009*b*), 1-(3-chlorophenyl)-3-(2,6-dichlorobenzoyl)thiourea (Khawar Rauf *et al.*, 2009*d*), 1-(3-chlorobenzoyl)-3-(2,3-dimethylphenyl)thiourea (Khawar Rauf *et al.*, 2009*c*), 1-(2,6-dichlorobenzoyl)-3-(2,3,5,6-tetrachlorophenyl)thiourea (Khawar Rauf *et al.*, 2009*a*), *N*'-(4-chlorobenzoyl)-*N*,*N*-diphenylthiourea (Arslan *et al.*, 2003a), 1-(2-chloro-benzoyl)-3-*p*-tolyl-thiourea (Arslan *et al.*, 2004), *N*,*N*-dimethyl-*N*-(2-chlorobenzoyl)thiourea (Arslan *et al.*, 2006), *o*-ethylbenzoylthiocarbamate (Arslan *et al.*, 2007*a*), 2-chloro-*N*-(diethylcarbamothioyl)benzamide (Arslan *et al.*, 2007*b*). The other bond lengths in (I) show normal values (Allen *et al.*, 1987).

The conformation of the title molecule with respect to the thiocarbonyl and carbonyl moieties is twisted, as reflected by the C101—N11—C108—O1, C108—N11—C101—S1, C108—N11—C101—N12, C201—N21—C208—O2, C208—N21—C201—S2, and C208—N21—C201—N22 torsion angles of 11.9 (5), 110.7 (3), -69.6 (4), -13.6 (5), -109.6 (3), and 70.5 (4)°, respectively. In addition, the difference in the torsion angles can be attributed to the different conformations of the two independent molecules.

The two di-*n*-propyl groups in independent molecules A (atom numbering 1xx) are twisted in a + and - antiperiplanar conformation with -179.9 (3)° and 178.2 (3)°. In the independent molecule B (atom numbering 2xx), one di-*n*-propyl group is twisted in a - antiperiplanar conformation with -179.1 (3)° and the other di-*n*-propyl group adopts a - synclinal conformation with -56.7 (4)°.

The phenyl rings and central thiourea S1—N11—N12—C101 [largest dev. 0.002 (3) Å for C101] and S2—N21—N22—C201 [largest dev. -0.001 (3) Å for C201] fragments are each essentially planar. The dihedral angle between the 4-bromophenyl ring and the plane S1/N11/N12/C101 is 84.88 (15)°, and the dihedral angle between the 4-bromophenyl ring and the plane S2/N21/N22/C201 is 82.53 (16)°.

The molecules of title compound are linked by paired N—H···S hydrogen bonds into centrosymmetric dimers. Details of the symmetry codes and hydrogen bonding are given in Table 1 and Fig. 2.

### Experimental

The title compound was prepared with a procedure similar to that reported in the literature (Arslan *et al.*, 2003*b*; Özer *et al.*, 2009). A solution of 4-bromobenzoyl chloride (0.01 mol) in acetone (50 ml) was added dropwise to a suspension of potassium thiocyanate (0.01 mol) in acetone (30 ml) (Fig. 3). The reaction mixture was heated under reflux for 30 min, and then cooled to room temperature. A solution of di-*n*-propylamine (0.01 mol) in acetone (10 ml) was added and the resulting mixture was stirred for 2 h. Hydrochloric acid (0.1 N, 300 ml) was added to the solution, which was then filtered. The solid product was washed with water and purifed by recrystallization from an ethanol–dichloromethane mixture (1:2). Anal. Calcd. for C_14_H_19_N_2_OSBr: C, 48.9; H, 5.6; N, 8.2. Found: C, 48.7; H, 5.4; N, 8.4%.

### Refinement

H atoms were clearly identified in difference syntheses. H atoms attached to nitrogens were located from a difference Fourier map and refined freely. The rest H atoms refined at idealized positions riding on the C atoms with C—H = 0.95–0.99 Å, and *U*_iso_(H) = 1.5*U*_eq_(C) for methyl H atoms and 1.2*U*_eq_(C) for other H atoms.

All CH_3_ H atoms were allowed to rotate but not to tip. For C203 and C204 neither anisotropic refinement nor split model provided successful results, so an isotropic model was used that gave sensible geometries but some electron density residuals nearby.

### Crystal data

**Table:**

C_14_H_19_BrN_2_OS	*F*(000) = 1408
*M**_r_* = 343.28	*D*_x_ = 1.454 Mg m^−^^3^
Monoclinic, *P*2_1_/*c*	Mo *K*α radiation, λ = 0.71073 Å
Hall symbol: -P 2ybc	Cell parameters from 948 reflections
*a* = 21.104 (3) Å	θ = 2.5–26.5°
*b* = 9.6940 (12) Å	µ = 2.75 mm^−^^1^
*c* = 16.208 (2) Å	*T* = 120 K
β = 108.956 (3)°	Needle, colourless
*V* = 3135.9 (7) Å^3^	0.48 × 0.18 × 0.17 mm
*Z* = 8	

### Data collection

**Table:**

Bruker SMART APEX diffractometer	7470 independent reflections
Radiation source: sealed tube	4686 reflections with *I* > 2σ(*I*)
graphite	*R*_int_ = 0.074
φ and ω scans	θ_max_ = 27.9°, θ_min_ = 2.0°
Absorption correction: multi-scan (*SADABS*; Sheldrick, 2004)	*h* = −27→27
*T*_min_ = 0.352, *T*_max_ = 0.652	*k* = −12→12
27091 measured reflections	*l* = −21→21

### Refinement

**Table:**

Refinement on *F*^2^	Primary atom site location: structure-invariant direct methods
Least-squares matrix: full	Secondary atom site location: difference Fourier map
*R*[*F*^2^ > 2σ(*F*^2^)] = 0.043	Hydrogen site location: difference Fourier map
*wR*(*F*^2^) = 0.094	H atoms treated by a mixture of independent and constrained refinement
*S* = 0.97	*w* = 1/[σ^2^(*F*_o_^2^) + (0.0334*P*)^2^] where *P* = (*F*_o_^2^ + 2*F*_c_^2^)/3
7470 reflections	(Δ/σ)_max_ = 0.001
340 parameters	Δρ_max_ = 1.70 e Å^−^^3^
2 restraints	Δρ_min_ = −0.71 e Å^−^^3^

### Special details

**Table:**

Geometry. All esds (except the esd in the dihedral angle between two l.s. planes) are estimated using the full covariance matrix. The cell esds are taken into account individually in the estimation of esds in distances, angles and torsion angles; correlations between esds in cell parameters are only used when they are defined by crystal symmetry. An approximate (isotropic) treatment of cell esds is used for estimating esds involving l.s. planes.
Refinement. Refinement of F^2^ against ALL reflections. The weighted R-factor wR and goodness of fit S are based on F^2^, conventional R-factors R are based on F, with F set to zero for negative F^2^. The threshold expression of F^2^ > 2sigma(F^2^) is used only for calculating R-factors(gt) etc. and is not relevant to the choice of reflections for refinement. R-factors based on F^2^ are statistically about twice as large as those based on F, and R- factors based on ALL data will be even larger.

### Fractional atomic coordinates and isotropic or equivalent isotropic displacement parameters (Å^2^)

**Table:**

	*x*	*y*	*z*	*U*_iso_*/*U*_eq_	
Br1	0.721214 (18)	1.21378 (4)	0.60004 (2)	0.03477 (11)	
S1	0.83552 (4)	0.37951 (8)	0.86296 (5)	0.02170 (19)	
O1	0.93367 (10)	0.7028 (2)	0.81254 (14)	0.0262 (5)	
N11	0.84288 (12)	0.5668 (3)	0.74656 (17)	0.0183 (6)	
H1	0.7986 (4)	0.576 (3)	0.735 (2)	0.028 (7)*	
N12	0.92646 (12)	0.4005 (3)	0.78106 (16)	0.0204 (6)	
C101	0.87200 (14)	0.4481 (3)	0.79479 (19)	0.0184 (7)	
C102	0.94737 (15)	0.4434 (3)	0.7063 (2)	0.0236 (7)	
H10A	0.9965	0.4320	0.7216	0.028*	
H10B	0.9368	0.5423	0.6940	0.028*	
C103	0.91272 (16)	0.3599 (4)	0.6251 (2)	0.0277 (8)	
H10C	0.9227	0.2608	0.6378	0.033*	
H10D	0.8637	0.3724	0.6093	0.033*	
C104	0.93463 (18)	0.4015 (4)	0.5486 (2)	0.0384 (10)	
H10E	0.9111	0.3449	0.4978	0.058*	
H10F	0.9831	0.3877	0.5635	0.058*	
H10G	0.9239	0.4990	0.5349	0.058*	
C105	0.96618 (15)	0.2880 (3)	0.8356 (2)	0.0263 (8)	
H10H	0.9928	0.2415	0.8034	0.032*	
H10I	0.9353	0.2190	0.8467	0.032*	
C106	1.01386 (16)	0.3413 (4)	0.9236 (2)	0.0321 (9)	
H10J	0.9868	0.3788	0.9581	0.039*	
H10K	1.0402	0.2627	0.9564	0.039*	
C107	1.06118 (17)	0.4506 (4)	0.9145 (2)	0.0366 (9)	
H10L	1.0897	0.4801	0.9726	0.055*	
H10M	1.0356	0.5298	0.8831	0.055*	
H10N	1.0893	0.4136	0.8820	0.055*	
C108	0.87557 (15)	0.6931 (3)	0.76526 (19)	0.0190 (7)	
C109	0.83589 (14)	0.8153 (3)	0.7228 (2)	0.0177 (7)	
C110	0.85099 (15)	0.9410 (3)	0.7660 (2)	0.0206 (7)	
H11A	0.8851	0.9452	0.8212	0.025*	
C111	0.81746 (15)	1.0591 (3)	0.7303 (2)	0.0235 (7)	
H11B	0.8271	1.1444	0.7606	0.028*	
C112	0.76883 (16)	1.0505 (3)	0.6482 (2)	0.0231 (7)	
C113	0.75382 (16)	0.9288 (3)	0.6034 (2)	0.0242 (7)	
H11C	0.7211	0.9258	0.5471	0.029*	
C114	0.78710 (15)	0.8097 (3)	0.6416 (2)	0.0217 (7)	
H11D	0.7764	0.7240	0.6118	0.026*	
Br2	0.784401 (18)	−0.25845 (4)	0.91522 (2)	0.03427 (11)	
S2	0.67033 (4)	0.57023 (9)	0.65301 (5)	0.02282 (19)	
O2	0.57058 (10)	0.2585 (2)	0.71095 (15)	0.0286 (5)	
N21	0.66312 (12)	0.3906 (3)	0.77447 (17)	0.0205 (6)	
H2	0.7080 (3)	0.387 (3)	0.7894 (19)	0.028 (7)*	
N22	0.58075 (12)	0.5590 (3)	0.73746 (17)	0.0211 (6)	
C201	0.63477 (15)	0.5074 (3)	0.7239 (2)	0.0198 (7)	
C202	0.53743 (15)	0.6593 (4)	0.6759 (2)	0.0269 (8)	
H20A	0.5640	0.7136	0.6470	0.032*	
H20B	0.5167	0.7236	0.7072	0.032*	
C203	0.4815 (2)	0.5730 (5)	0.6062 (3)	0.0590 (12)*	
H20C	0.5028	0.5082	0.5760	0.071*	
H20D	0.4557	0.5184	0.6359	0.071*	
C204	0.4363 (2)	0.6658 (5)	0.5424 (3)	0.0759 (15)*	
H20E	0.4023	0.6114	0.4990	0.114*	
H20F	0.4620	0.7201	0.5133	0.114*	
H20G	0.4143	0.7280	0.5723	0.114*	
C205	0.55988 (16)	0.5263 (3)	0.8138 (2)	0.0265 (8)	
H20H	0.5752	0.4323	0.8348	0.032*	
H20I	0.5104	0.5281	0.7965	0.032*	
C206	0.58920 (17)	0.6295 (4)	0.8863 (2)	0.0324 (9)	
H20J	0.5752	0.7236	0.8643	0.039*	
H20K	0.6387	0.6253	0.9045	0.039*	
C207	0.56663 (19)	0.6012 (4)	0.9654 (2)	0.0459 (11)	
H20L	0.5861	0.6705	1.0105	0.069*	
H20M	0.5817	0.5092	0.9885	0.069*	
H20N	0.5176	0.6059	0.9477	0.069*	
C208	0.62915 (15)	0.2660 (3)	0.7571 (2)	0.0211 (7)	
C209	0.66820 (15)	0.1411 (3)	0.7984 (2)	0.0204 (7)	
C210	0.65274 (16)	0.0172 (3)	0.7536 (2)	0.0224 (7)	
H21A	0.6184	0.0143	0.6985	0.027*	
C211	0.68704 (16)	−0.1023 (3)	0.7884 (2)	0.0238 (7)	
H21B	0.6773	−0.1870	0.7574	0.029*	
C212	0.73567 (16)	−0.0953 (3)	0.8693 (2)	0.0243 (8)	
C213	0.75084 (16)	0.0257 (3)	0.9163 (2)	0.0253 (8)	
H21C	0.7836	0.0272	0.9727	0.030*	
C214	0.71721 (15)	0.1455 (3)	0.8796 (2)	0.0234 (7)	
H21D	0.7278	0.2304	0.9102	0.028*	

### Atomic displacement parameters (Å^2^)

**Table:**

	*U*^11^	*U*^22^	*U*^33^	*U*^12^	*U*^13^	*U*^23^
Br1	0.0403 (2)	0.0273 (2)	0.0392 (2)	0.01111 (16)	0.01621 (18)	0.01217 (16)
S1	0.0196 (4)	0.0208 (5)	0.0249 (4)	−0.0010 (3)	0.0076 (4)	0.0011 (3)
O1	0.0162 (12)	0.0206 (13)	0.0367 (14)	−0.0004 (9)	0.0017 (11)	−0.0033 (10)
N11	0.0116 (13)	0.0164 (15)	0.0267 (15)	−0.0002 (11)	0.0060 (12)	0.0009 (12)
N12	0.0174 (14)	0.0166 (15)	0.0272 (15)	0.0022 (11)	0.0073 (12)	0.0000 (12)
C101	0.0158 (16)	0.0162 (17)	0.0204 (16)	−0.0021 (13)	0.0017 (13)	−0.0050 (13)
C102	0.0209 (17)	0.0221 (19)	0.0317 (19)	0.0003 (14)	0.0137 (15)	−0.0016 (15)
C103	0.0283 (19)	0.025 (2)	0.0284 (19)	−0.0011 (15)	0.0067 (16)	−0.0041 (15)
C104	0.039 (2)	0.048 (3)	0.029 (2)	−0.0021 (18)	0.0125 (18)	−0.0080 (18)
C105	0.0205 (17)	0.022 (2)	0.035 (2)	0.0064 (14)	0.0067 (15)	0.0009 (15)
C106	0.0219 (18)	0.033 (2)	0.038 (2)	0.0077 (16)	0.0042 (17)	0.0048 (17)
C107	0.0246 (19)	0.040 (2)	0.042 (2)	0.0011 (17)	0.0066 (17)	−0.0095 (18)
C108	0.0212 (18)	0.0187 (18)	0.0202 (17)	−0.0016 (14)	0.0112 (15)	−0.0038 (13)
C109	0.0157 (16)	0.0189 (18)	0.0224 (17)	−0.0002 (13)	0.0118 (14)	0.0021 (13)
C110	0.0195 (17)	0.0199 (19)	0.0227 (17)	−0.0040 (14)	0.0074 (14)	−0.0020 (14)
C111	0.0244 (18)	0.0206 (19)	0.0284 (18)	0.0001 (14)	0.0126 (15)	−0.0041 (15)
C112	0.0275 (18)	0.0176 (19)	0.0282 (19)	0.0062 (14)	0.0147 (16)	0.0082 (14)
C113	0.0221 (18)	0.029 (2)	0.0201 (17)	0.0004 (15)	0.0053 (14)	0.0002 (15)
C114	0.0231 (18)	0.0190 (19)	0.0259 (18)	−0.0035 (13)	0.0119 (15)	−0.0024 (14)
Br2	0.0420 (2)	0.0286 (2)	0.0375 (2)	0.01163 (17)	0.02020 (18)	0.01355 (17)
S2	0.0195 (4)	0.0242 (5)	0.0263 (4)	0.0003 (3)	0.0095 (4)	−0.0001 (4)
O2	0.0177 (12)	0.0221 (14)	0.0438 (14)	−0.0028 (10)	0.0068 (11)	−0.0020 (11)
N21	0.0155 (14)	0.0159 (15)	0.0304 (16)	0.0008 (11)	0.0076 (13)	−0.0001 (12)
N22	0.0177 (14)	0.0178 (15)	0.0284 (15)	0.0032 (11)	0.0085 (12)	−0.0002 (12)
C201	0.0156 (16)	0.0183 (18)	0.0231 (17)	−0.0028 (13)	0.0030 (14)	−0.0053 (14)
C202	0.0212 (18)	0.033 (2)	0.0246 (19)	0.0117 (15)	0.0048 (15)	0.0006 (15)
C205	0.0226 (18)	0.026 (2)	0.037 (2)	0.0044 (14)	0.0175 (16)	0.0030 (16)
C206	0.031 (2)	0.040 (2)	0.0271 (19)	−0.0021 (17)	0.0097 (16)	−0.0020 (17)
C207	0.044 (2)	0.062 (3)	0.036 (2)	0.002 (2)	0.020 (2)	−0.001 (2)
C208	0.0196 (17)	0.0200 (19)	0.0270 (17)	0.0001 (14)	0.0121 (15)	−0.0032 (14)
C209	0.0196 (17)	0.0163 (18)	0.0287 (18)	−0.0016 (13)	0.0123 (15)	−0.0001 (14)
C210	0.0209 (18)	0.024 (2)	0.0232 (17)	−0.0030 (14)	0.0080 (15)	−0.0005 (14)
C211	0.0298 (19)	0.0175 (19)	0.0277 (19)	0.0000 (14)	0.0140 (16)	−0.0014 (14)
C212	0.0262 (18)	0.021 (2)	0.0311 (19)	0.0042 (15)	0.0171 (16)	0.0084 (15)
C213	0.0262 (19)	0.025 (2)	0.0249 (18)	−0.0016 (15)	0.0079 (15)	0.0005 (15)
C214	0.0271 (19)	0.0182 (19)	0.0278 (19)	−0.0034 (14)	0.0130 (16)	−0.0031 (15)

### Geometric parameters (Å, °)

**Table:**

Br1—C112	1.901 (3)	Br2—C212	1.902 (3)
S1—C101	1.676 (3)	S2—C201	1.678 (3)
O1—C108	1.220 (3)	O2—C208	1.221 (3)
N11—C108	1.389 (4)	N21—C208	1.386 (4)
N11—C101	1.415 (4)	N21—C201	1.412 (4)
N11—H1	0.896 (15)	N21—H2	0.899 (14)
N12—C101	1.323 (4)	N22—C201	1.327 (4)
N12—C102	1.478 (4)	N22—C205	1.476 (4)
N12—C105	1.481 (4)	N22—C202	1.477 (4)
C102—C103	1.515 (4)	C202—C203	1.582 (5)
C102—H10A	0.9900	C202—H20A	0.9900
C102—H10B	0.9900	C202—H20B	0.9900
C103—C104	1.512 (4)	C203—C204	1.465 (6)
C103—H10C	0.9900	C203—H20C	0.9900
C103—H10D	0.9900	C203—H20D	0.9900
C104—H10E	0.9800	C204—H20E	0.9800
C104—H10F	0.9800	C204—H20F	0.9800
C104—H10G	0.9800	C204—H20G	0.9800
C105—C106	1.543 (4)	C205—C206	1.515 (4)
C105—H10H	0.9900	C205—H20H	0.9900
C105—H10I	0.9900	C205—H20I	0.9900
C106—C107	1.495 (5)	C206—C207	1.529 (4)
C106—H10J	0.9900	C206—H20J	0.9900
C106—H10K	0.9900	C206—H20K	0.9900
C107—H10L	0.9800	C207—H20L	0.9800
C107—H10M	0.9800	C207—H20M	0.9800
C107—H10N	0.9800	C207—H20N	0.9800
C108—C109	1.484 (4)	C208—C209	1.495 (4)
C109—C114	1.384 (4)	C209—C214	1.385 (4)
C109—C110	1.390 (4)	C209—C210	1.387 (4)
C110—C111	1.371 (4)	C210—C211	1.386 (4)
C110—H11A	0.9500	C210—H21A	0.9500
C111—C112	1.394 (4)	C211—C212	1.380 (4)
C111—H11B	0.9500	C211—H21B	0.9500
C112—C113	1.367 (4)	C212—C213	1.379 (4)
C113—C114	1.388 (4)	C213—C214	1.390 (4)
C113—H11C	0.9500	C213—H21C	0.9500
C114—H11D	0.9500	C214—H21D	0.9500
			
C108—N11—C101	120.0 (2)	C208—N21—C201	119.2 (3)
C108—N11—H1	112 (2)	C208—N21—H2	117 (2)
C101—N11—H1	116 (2)	C201—N21—H2	114 (2)
C101—N12—C102	123.2 (3)	C201—N22—C205	124.3 (3)
C101—N12—C105	120.7 (3)	C201—N22—C202	120.9 (3)
C102—N12—C105	115.8 (2)	C205—N22—C202	114.8 (2)
N12—C101—N11	115.8 (3)	N22—C201—N21	115.6 (3)
N12—C101—S1	125.7 (2)	N22—C201—S2	125.2 (2)
N11—C101—S1	118.5 (2)	N21—C201—S2	119.3 (2)
N12—C102—C103	111.9 (2)	N22—C202—C203	106.8 (3)
N12—C102—H10A	109.2	N22—C202—H20A	110.4
C103—C102—H10A	109.2	C203—C202—H20A	110.4
N12—C102—H10B	109.2	N22—C202—H20B	110.4
C103—C102—H10B	109.2	C203—C202—H20B	110.4
H10A—C102—H10B	107.9	H20A—C202—H20B	108.6
C104—C103—C102	112.3 (3)	C204—C203—C202	110.1 (4)
C104—C103—H10C	109.1	C204—C203—H20C	109.6
C102—C103—H10C	109.1	C202—C203—H20C	109.6
C104—C103—H10D	109.1	C204—C203—H20D	109.6
C102—C103—H10D	109.1	C202—C203—H20D	109.6
H10C—C103—H10D	107.9	H20C—C203—H20D	108.2
C103—C104—H10E	109.5	C203—C204—H20E	109.5
C103—C104—H10F	109.5	C203—C204—H20F	109.5
H10E—C104—H10F	109.5	H20E—C204—H20F	109.5
C103—C104—H10G	109.5	C203—C204—H20G	109.5
H10E—C104—H10G	109.5	H20E—C204—H20G	109.5
H10F—C104—H10G	109.5	H20F—C204—H20G	109.5
N12—C105—C106	112.2 (3)	N22—C205—C206	110.5 (3)
N12—C105—H10H	109.2	N22—C205—H20H	109.5
C106—C105—H10H	109.2	C206—C205—H20H	109.5
N12—C105—H10I	109.2	N22—C205—H20I	109.5
C106—C105—H10I	109.2	C206—C205—H20I	109.5
H10H—C105—H10I	107.9	H20H—C205—H20I	108.1
C107—C106—C105	113.8 (3)	C205—C206—C207	111.9 (3)
C107—C106—H10J	108.8	C205—C206—H20J	109.2
C105—C106—H10J	108.8	C207—C206—H20J	109.2
C107—C106—H10K	108.8	C205—C206—H20K	109.2
C105—C106—H10K	108.8	C207—C206—H20K	109.2
H10J—C106—H10K	107.7	H20J—C206—H20K	107.9
C106—C107—H10L	109.5	C206—C207—H20L	109.5
C106—C107—H10M	109.5	C206—C207—H20M	109.5
H10L—C107—H10M	109.5	H20L—C207—H20M	109.5
C106—C107—H10N	109.5	C206—C207—H20N	109.5
H10L—C107—H10N	109.5	H20L—C207—H20N	109.5
H10M—C107—H10N	109.5	H20M—C207—H20N	109.5
O1—C108—N11	122.1 (3)	O2—C208—N21	122.1 (3)
O1—C108—C109	122.0 (3)	O2—C208—C209	121.8 (3)
N11—C108—C109	115.9 (3)	N21—C208—C209	116.2 (3)
C114—C109—C110	119.4 (3)	C214—C209—C210	120.0 (3)
C114—C109—C108	122.9 (3)	C214—C209—C208	122.3 (3)
C110—C109—C108	117.6 (3)	C210—C209—C208	117.7 (3)
C111—C110—C109	121.1 (3)	C211—C210—C209	120.5 (3)
C111—C110—H11A	119.4	C211—C210—H21A	119.7
C109—C110—H11A	119.4	C209—C210—H21A	119.7
C110—C111—C112	118.2 (3)	C212—C211—C210	118.4 (3)
C110—C111—H11B	120.9	C212—C211—H21B	120.8
C112—C111—H11B	120.9	C210—C211—H21B	120.8
C113—C112—C111	122.0 (3)	C213—C212—C211	122.3 (3)
C113—C112—Br1	120.0 (2)	C213—C212—Br2	119.5 (3)
C111—C112—Br1	117.9 (2)	C211—C212—Br2	118.1 (2)
C112—C113—C114	119.0 (3)	C212—C213—C214	118.7 (3)
C112—C113—H11C	120.5	C212—C213—H21C	120.7
C114—C113—H11C	120.5	C214—C213—H21C	120.7
C109—C114—C113	120.2 (3)	C209—C214—C213	120.1 (3)
C109—C114—H11D	119.9	C209—C214—H21D	120.0
C113—C114—H11D	119.9	C213—C214—H21D	120.0
			
C102—N12—C101—N11	−14.7 (4)	C205—N22—C201—N21	15.9 (4)
C105—N12—C101—N11	172.2 (3)	C202—N22—C201—N21	−165.8 (3)
C102—N12—C101—S1	165.0 (2)	C205—N22—C201—S2	−164.0 (2)
C105—N12—C101—S1	−8.1 (4)	C202—N22—C201—S2	14.2 (4)
C108—N11—C101—N12	−69.6 (4)	C208—N21—C201—N22	70.4 (4)
C108—N11—C101—S1	110.7 (3)	C208—N21—C201—S2	−109.6 (3)
C101—N12—C102—C103	−84.6 (4)	C201—N22—C202—C203	91.1 (3)
C105—N12—C102—C103	88.8 (3)	C205—N22—C202—C203	−90.5 (3)
N12—C102—C103—C104	−179.1 (3)	N22—C202—C203—C204	−179.9 (3)
C101—N12—C105—C106	−81.1 (4)	C201—N22—C205—C206	91.7 (4)
C102—N12—C105—C106	105.3 (3)	C202—N22—C205—C206	−86.6 (3)
N12—C105—C106—C107	−56.6 (4)	N22—C205—C206—C207	178.1 (3)
C101—N11—C108—O1	11.9 (4)	C201—N21—C208—O2	−13.6 (4)
C101—N11—C108—C109	−169.3 (2)	C201—N21—C208—C209	166.7 (3)
O1—C108—C109—C114	147.1 (3)	O2—C208—C209—C214	−146.2 (3)
N11—C108—C109—C114	−31.8 (4)	N21—C208—C209—C214	33.6 (4)
O1—C108—C109—C110	−30.3 (4)	O2—C208—C209—C210	32.2 (4)
N11—C108—C109—C110	150.9 (3)	N21—C208—C209—C210	−148.1 (3)
C114—C109—C110—C111	1.3 (4)	C214—C209—C210—C211	−1.5 (4)
C108—C109—C110—C111	178.7 (3)	C208—C209—C210—C211	−179.9 (3)
C109—C110—C111—C112	−1.6 (4)	C209—C210—C211—C212	1.4 (4)
C110—C111—C112—C113	0.4 (5)	C210—C211—C212—C213	0.3 (5)
C110—C111—C112—Br1	178.7 (2)	C210—C211—C212—Br2	−178.1 (2)
C111—C112—C113—C114	1.2 (5)	C211—C212—C213—C214	−1.9 (5)
Br1—C112—C113—C114	−177.1 (2)	Br2—C212—C213—C214	176.5 (2)
C110—C109—C114—C113	0.3 (4)	C210—C209—C214—C213	−0.1 (4)
C108—C109—C114—C113	−177.0 (3)	C208—C209—C214—C213	178.2 (3)
C112—C113—C114—C109	−1.6 (4)	C212—C213—C214—C209	1.8 (5)

### Hydrogen-bond geometry (Å, °)

**Table:**

*D*—H···*A*	*D*—H	H···*A*	*D*···*A*	*D*—H···*A*
N11—H1···S2	0.90 (2)	2.60 (2)	3.460 (3)	161 (3)
N21—H2···S1	0.90 (1)	2.57 (2)	3.452 (3)	169 (2)
